# Reoxidation of IF Steel Caused by Cr_2_O_3_-Based Stuffing Sand and Its Optimization

**DOI:** 10.3390/ma18173945

**Published:** 2025-08-22

**Authors:** Chenhui Wu, Youquan Peng, Jiqing Zhang, Jianhua Zhang, Xin Xie

**Affiliations:** 1 Automobile & Household Appliances Steels Division, Pangang Group Research Institute Co., Ltd., Panzhihua 617000, China; xiexin_xc@126.com; 2 Pangang Group Xichang Steel Vanadium Co., Ltd., Xichang 615032, China; pyq_xc@126.com (Y.P.); zjh_xc@126.com (J.Z.); 3 School of Materials Science and Engineering, University of Science and Technology Beijing, Beijing 100083, China; m202440031@xs.ustb.edu.cn

**Keywords:** IF steel, stuffing sand, reoxidation, burning loss, T.[O]

## Abstract

Stuffing sand, as a critical auxiliary material, plays an important role in ladle teeming during the continuous casting process and is closely related to steel cleanliness. Based on thermodynamic calculations, a melting test in a vacuum induction furnace, and industrial statistical data analysis, the reoxidation of IF steel caused by conventional Cr_2_O_3_-based stuffing sand was investigated. The results show that Cr_2_O_3_-based stuffing sand is one of the main factors resulting in the reoxidation of IF steel. [Al] and [Ti] in IF steel can be oxidized by FeO, Cr_2_O_3_, and SiO_2_ from the Cr_2_O_3_-based stuffing sand, which leads to the mass burning loss of [Al] and [Ti], thus resulting in the deterioration of steel cleanliness. After reoxidation caused by Cr_2_O_3_-based stuffing sand, the [Cr] content in IF steel increases by 70 ppm on average. To avoid reoxidation pollution by conventional Cr_2_O_3_-based stuffing sand, a new kind of Al_2_O_3_-based stuffing sand with low reactivity was developed and applied in industrial production. After adopting this new kind of stuffing sand, the burning loss of [Al] and [Ti] decreases by 41.3% and 24.2%, respectively, and the total oxygen content (T.[O]) of the steel in the tundish decreases by 35.2% compared with the conventional Cr_2_O_3_-based stuffing sand.

## 1. Introduction

Steel reoxidation has a detrimental influence on production process stability and final product quality [1] as it increases the number, density, and size of nonmetallic inclusions in steel. This, in turn, leads to nozzle clogging problems and product defects [2,3,4]. Therefore, steel reoxidation should be reduced as much as possible, particularly for steel requiring high cleanliness.

Steel reoxidation is caused by multiple factors, including oxygen in air, reducible oxides in slag, and refractories, etc., which have been widely investigated in the past. Many studies [5,6,7] focusing on the influence of oxygen in air on steel reoxidation during the refining and continuous casting process have been conducted. Sasai et al. [5] successfully predicted the amount of steel oxidation caused by oxygen in air in the tundish using a reaction rate model. Wang et al. [6] were the first to quantify the influence of oxygen partial pressures on the growth of Al_2_O_3_ at steel/gas interfaces through in situ observation using a confocal scanning laser microscope. Wang et al. [7] calculated the composition of inclusions due to oxygen in air and qualified the integrated free surface area and air-to-steel volume ratio during pouring by developing a calculation model. Slag and refractories are other important factors that result in reoxidation, and they have been investigated in many reports [8,9,10,11,12,13,14]. Among these studies, Park et al. [10] and Yan et al. [12] found that MnO, FeO, and SiO_2_ in slag play important roles in steel reoxidation, and Yan et al. also concluded that CaO-Al_2_O_3_ slag with a higher FeO + MnO content provided more oxygen to molten steel than CaO-Al_2_O_3_-SiO_2_ slag. Furthermore, Yan et al. [12] investigated steel reoxidation by two different types of gunning mass (GM) and found that the oxidation capacity of MgO GM was stronger than that of Al_2_O_3_ GM due to the higher content of SiO_2_ + FeO in MgO GM. Qin et al. [11] and Cho et al. [14] studied reoxidation of IF steel and found that the (pct CaO)/(pct Al_2_O_3_) ratio of slag and slag reoxidation for different processing times could significantly affect steel reoxidation. For Ca-treated steels, reoxidation occurs more easily due to the addition of Ca with a high chemical reactivity, which significantly affects steel cleanliness and the effect of Ca treatment. Therefore, many studies on the reoxidation of Ca-treated steels have been conducted with experiments [15,16,17] or kinetic models [18].

Stuffing sand is a critical metallurgical auxiliary material, which is filled in the upper nozzle of the ladle and isolates the liquid steel and the slide gate [19,20]. Currently, Cr_2_O_3_-based stuffing sand has been widely applied in plants due to its low cost and high self-opening rate in the ladle. During our investigation, it was found that Cr_2_O_3_-based stuffing sand was a main factor resulting in steel reoxidation. However, this has not yet been reported in previous studies that have mainly focused on steel reoxidation caused by oxygen in air, slag, and refractories, etc. Interstitial-free (IF) steels are often used for producing external automotive panels. Reoxidation of IF steel occurs easily due to alloying [Al] and [Ti] elements, described by the following reactions: [Al] + [O] → (Al_2_O_3_) and [Ti] + [O] → (TiO_2_) [21]. Massive nonmetallic inclusions (Al_2_O_3_, TiO_2_) with a large size form during the reoxidation process, which results in burning loss of [Al] and [Ti] and ultimately leads to silver defects in the final cold-rolling automotive panels [22,23].

To elucidate the influence of stuffing sand on steel oxidation, the reoxidation process of IF steel caused by conventional Cr_2_O_3_-based stuffing sand was first investigated in the present work through thermodynamic calculations, a melting test in a vacuum induction furnace, and industrial experiments. Subsequently, a new kind of Al_2_O_3_-based stuffing sand with low reactivity was then developed and applied for the first time. The main component of this newly developed Al_2_O_3_-based stuffing sand is Al_2_O_3_ (>80 wt%), which is significantly different from conventional Cr_2_O_3_-based stuffing sand, which has a main composition of Cr_2_O_3_ + FeO + SiO_2_ (>70 wt%). The adverse effect of conventional Cr_2_O_3_-based stuffing sand, which results in severe reoxidation, is largely eliminated by this new kind of stuffing sand, and the steel cleanliness is improved dramatically.

## 2. Experimental Section

### 2.1. Materials

Table 1 shows the main composition of IF steel (M3A35. Manufactured in Xichang, Sichuan, China), which is produced in a BOF (basic oxygen furnace)–RH (Ruhrstahl–Heraeus)–CC (continuous casting) process. The contents of [Al] and [Ti] in IF steel reach 0.03% and 0.06%, respectively.

Table 2 shows the composition of different types of stuffing sand (Manufactured in Xichang, Sichuan, China). The Cr_2_O_3_-based stuffing sand (1#) is the conventional type and has been widely used in industrial production. It is mainly composed of Cr_2_O_3_, SiO_2_, Al_2_O_3_, FeO, and MgO. To reduce the reoxidation influence of the conventional Cr_2_O_3_-based stuffing sand, a new kind of stuffing sand, Al_2_O_3-_based stuffing sand (2#–4#), was designed and investigated in the present work. The main components of the Al_2_O_3_-based stuffing sand are Al_2_O_3_ and SiO_2_.

### 2.2. Experimental Procedure

#### 2.2.1. Melting Test

**Figure 1:**
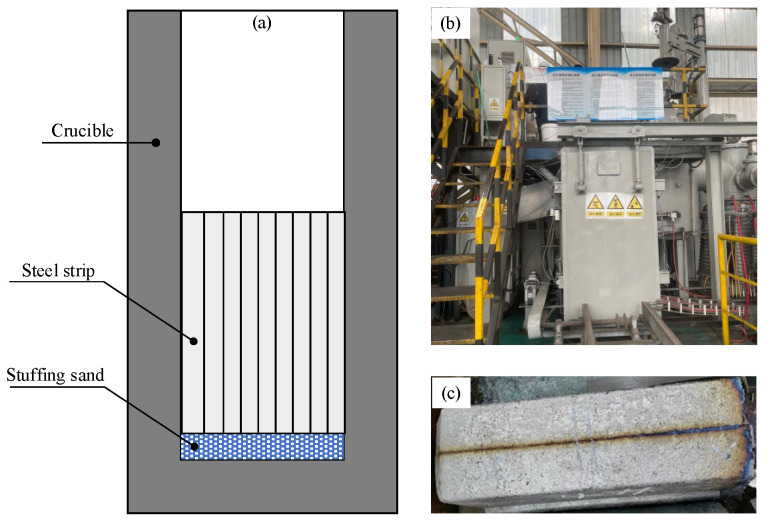
(**a**) Crucible filled with stuffing sand and steel strips, (**b**) 150 kg vacuum induction furnace, and (**c**) casting ingots.

To evaluate the influence of stuffing sand on the reoxidation of IF steel, melting tests were conducted in a 150 kg vacuum induction furnace. For each test, 37.2 g of stuffing sand was filled at the bottom of the crucible. IF steel slab taken from the steel plant is processed into strips, and 117 kg of steel strips are filled in the upper part of the crucible. Figure 1a shows the crucible filled with stuffing sand and steel strips. The weight ratio of the filled stuffing sand and steel strip in the test is equal to that of 70 kg stuffing sand and 220 t steel for a ladle in the practical production. The compositions of the stuffing sand and IF steel are listed in Table 1 and Table 2, respectively. During the test, the vacuum degree in the melting chamber of the vacuum induction furnace, as shown in Figure 1b, was firstly reduced to below 10 Pa, and then induction heat was introduced. The crucible temperature was maintained at 1600 °C for 40 min after all the steel strips had melted completely. During this process, the liquid steel could react with the stuffing sand sufficiently. After the heat preservation finished, the liquid steel in the crucible was cast directly into an ingot under vacuum conditions. Figure 1 shows the casting ingots.

#### 2.2.2. Determination of Refractoriness

**Figure 2:**
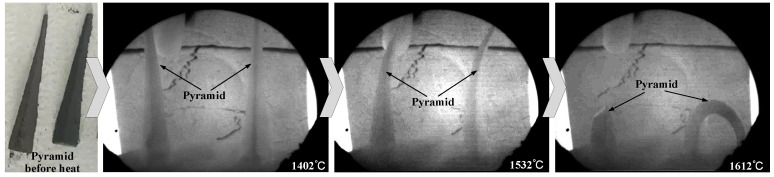
Morphology variation of the stuffing sand pyrometric cone during refractoriness test.

To determine the refractoriness of stuffing sands 1#–4# in Table 3, Pyrometric Cone Equivalent (PCE) tests were conducted. Pyrometric cones made of stuffing sand were prepared, and the cone size was 40 mm height × 8 mm base × 2 mm top. During testing, two identical pyrometric cones were installed on a refractory base at a 10° inclination with a spacing of 30 mm. The cones were then heated with the heating rate of 10 °C/min below 1000 °C and 3 °C/min above 1000 °C. With the continuous increase in temperature, the cones began to bend gradually as shown in Figure 2. The temperature at which the cone tips bent and touched the base was recorded as the final measured refractoriness result.

### 2.3. Methods of Detection and Analysis

#### 2.3.1. Methods of Detection

The main element content of steel samples obtained from the melting test and industrial production was determined using an X-ray fluorescence spectrometer (Manufactured by Thermo Fisher Scientific, Waltham, MA, USA). The total oxygen contents (T.[O]) of steel samples were analyzed by a TCH-600 oxygen/nitrogen/hydrogen analyzer (Manufactured by LECO Corporation, St. Joseph, MI, USA). 

#### 2.3.2. Methods of Analysis

Gibbs energy change (ΔG) of reactions between [Al] and [Ti] of IF steel and the main composition of the stuffing sand were calculated at 1550 °C using the Reaction Module of the FactSage 8.0 thermodynamic software. The FactPS and FToxid databases are selected during the calculation.

The Equilib Module of FactSage 8.0 was employed to quantitatively calculate the influence of stuffing sand on the reoxidation of IF steel at 1550 °C for the practical industry production process and at 1600 °C for the melting test process. The average weight of the filled stuffing sand and molten steel in a ladle is about 70 kg and 220 t, respectively. Therefore, the calculated reaction system for the practical industry production process contains 70 kg of Cr_2_O_3_-based stuffing sand and 220 t of IF steel. Table 3 and Table 4 show the composition of the stuffing sand and IF steel in the calculated reaction system for industry production, respectively. For the melting test process, the calculated reaction system contains two groups: 37.2 g of stuffing sand 1# and 117 kg of steel for Group A and 37.2 g of stuffing sand 2# and 117 kg of steel for Group B. The weight ratio of stuffing sand and steel in each group is equal to that of 70 kg stuffing sand and 220 t steel in the practical industry production. Table 5 and Table 6 shows the composition of the stuffing sand and IF steel in each group of the calculated reaction system for the melting test process.

## 3. Results and Discussion

### 3.1. Influence of Stuffing Sand on Reoxidation of Steel

#### 3.1.1. Analysis of Thermodynamic Calculation Results

Table 7 presents the Gibbs energy change (ΔG) of reactions between [Al] and [Ti] of IF steel and the main composition of the stuffing sand. The calculated ΔG values for Groups A and B indicate that both [Al] and [Ti] in IF steel can be oxidized by Cr_2_O_3_, SiO_2_, and FeO in the Cr_2_O_3_-based stuffing sand, with these components accounting for 76% of the total content. This means that most components of the stuffing sand can cause the reoxidation of [Al] and [Ti] in IF steel and thus lead to an adverse impact on the steel cleanliness. The absolute values of ΔG in Table 7 are in the order of (c) > (a) > (b) for Group A and (h) > (e) > (f) for Group B. This indicates that the oxidizability of the Cr_2_O_3_-based stuffing sand composition is in the order of FeO > Cr_2_O_3_ > SiO_2_. The absolute ΔG value of (j) is larger than that of (k), which indicates the [Al] in IF steel can be oxidized much more easily than [Ti].

In the practical production process, partial oxidation of [Al] and [Ti] inevitably occurs due to oxidizing agents such as atmospheric oxygen and oxidizing components within the slag, which leads to burning loss of [Al] and [Ti]. Massive inclusions of Al_2_O_3_ and TiO_2_ form during this burning loss process and thus largely affect the steel cleanliness. Consequently, the burning loss amount of [Al] and [Ti] from the end of RH to the tundish is usually regarded as an important index indicating the pollution of the steel caused by reoxidation and can be calculated as follows:

Δ*w*[Al] = *w*[Al]_RH_ − *w*[Al]_Tun_ (1)

Δ*w*[Ti] = *w*[Ti]_RH_ − *w*[Ti]_Tun_ (2)

where Δ*w*[Al] and Δ*w*[Ti] represent the burning loss amount of [Al] and [Ti]; *w*[Al]_RH_ and *w*[Ti]_RH_ represent the content of [Al] and [Ti] of steel at end of RH; and *w*[Al]_Tun_ and *w*[Ti]_Tun_ represent the content of [Al] and [Ti] of steel in the tundish. Larger values of Δ*w*[Al] and Δ*w*[Ti] represent a more severe pollution degree caused by reoxidation.

**Figure 3:**
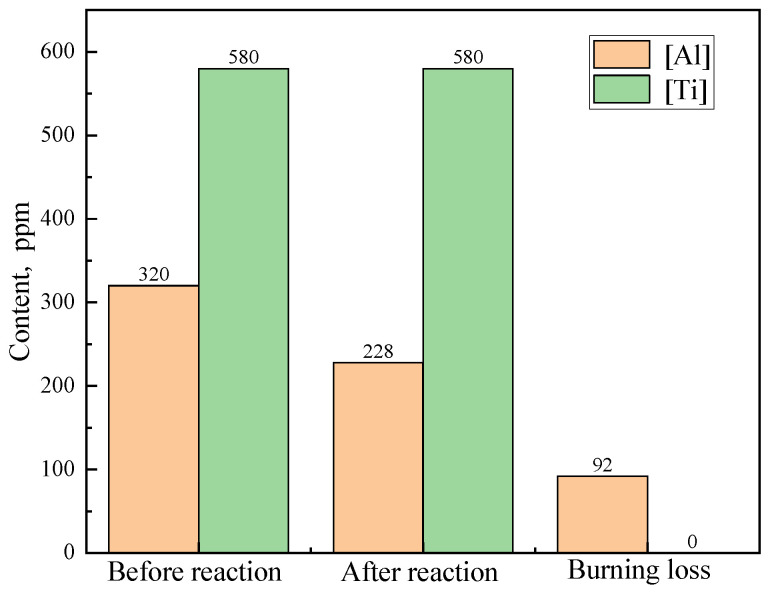
Variation in [Al] and [Ti] before and after the reaction.

Figure 3 presents the calculated variation in [Al] and [Ti] before and after the reaction of 70 kg of stuffing sand with 220 t of IF steel for one heat (the specific reaction system is shown in Table 3 and Table 4) and simultaneously shows the corresponding burning loss amount. The contents before and after the reaction in Figure 3 correspond to the contents of steel at end of RH and in the tundish in Equations (1) and (2). So, the burning loss in Figure 3 is equal to that the content before reaction minus the content after the reaction. Due to the oxidability of the stuffing sand, Δ*w*[Al] reaches 92 ppm. However, Δ*w*[Ti] is 0 ppm. This is because [Al] can be oxidized much easier than [Ti], as mentioned above, and the content of [Al] is still high (228 ppm) after the reaction between stuffing sand and steel. As a result, [Ti] can hardly be oxidized by the stuffing sand.

**Figure 4:**
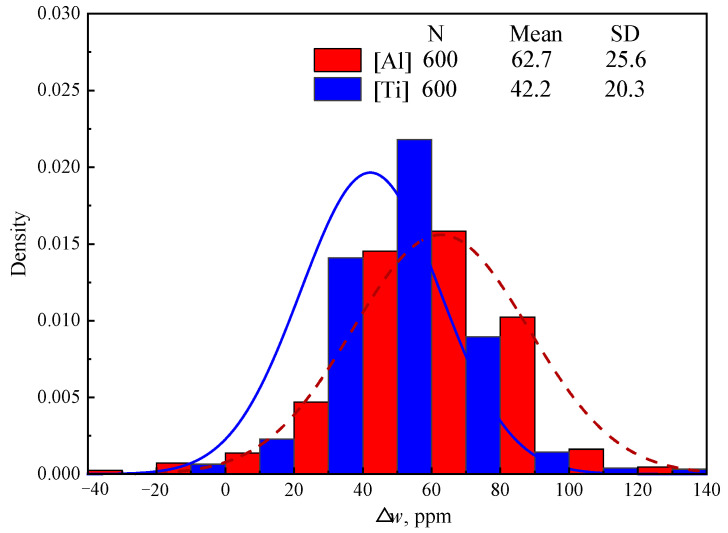
Distribution of the detected Δ*w*[Al] and Δ*w*[Ti] in industry production.

Figure 4 shows the detected Δ*w*[Al] and Δ*w*[Ti] of 600 heats in industry production, which was calculated based on the detected *w*[Al]_RH_, *w*[Ti]_RH_, *w*[Al]_Tun_, and *w*[Ti]_Tun_ of steel samples collected at the end of RH and in the tundish. “N”, “Mean” and “SD” in Figure 4 represent the number of samples, mean value of samples, and standard deviation of samples, respectively. Although the content of [Ti] in IF steel is twice than that of [Al] as shown in Table 1, Δ*w*[Ti] (average 42.2 ppm) is significantly lower than Δ*w*[Al] (average 62.7 ppm). This proves that [Al] can be oxidized much more easily than [Ti], which is consistent with the calculated results above in Group C in Table 7.

The calculated Δ*w*[Al] (shown in Figure 1) reaches 92 ppm, which is obviously larger than the detected result of 62.7 ppm in industry production (shown in Figure 4). This can be attributed to three reasons. Firstly, the calculated results in Figure 3 are at a thermodynamic equilibrium state, and all the filled stuffing sand reacts with IF steel completely. However, in industry production, some of the filled stuffing sand inevitably floats to ladle slag during BOF tapping, and some others adheres to the inside of the upper nozzle or shroud during steel ladle teeming. Consequently, only partially filled stuffing sand can enter the tundish and react with the liquid steel. Secondly, some of the stuffing sand that enters the tundish can easily float to the tundish slag due to its large particle size (0.15~1 mm), which depresses the reaction between stuffing sand and IF steel. Thirdly, the tundish slag is a CaO-Al_2_O_3_ type, and the Al_2_O_3_ content is more than 30%. The high content of Al_2_O_3_ in the tundish inhibits the reoxidation process of (M*_x_*O*_y_*) + [Al] → [M] + (Al_2_O_3_) (M*_x_*O*_y_* denotes FeO, Cr_2_O_3_, SiO_2_ of the Cr_2_O_3_-based stuffing sand) between stuffing sand and [Al] of the IF steel, which is helpful for decreasing Δ*w*[Al].

The calculated results in Figure 3 indicate that the [Ti] in IF steel cannot be oxidized by the Cr_2_O_3_-based stuffing sand. However, the detected results in industry production in Figure 4 show that the average practical Δ*w*[Ti] reaches 42.2 ppm. This significant difference between the detected and the calculated results can be explained by two reasons. Firstly, although the high content of Al_2_O_3_ in tundish slag inhibits the reaction of (M*_x_*O*_y_*) + [Al] → [M] + (Al_2_O_3_), the reaction of (M*_x_*O*_y_*) + [Ti] → [M] + (TiO_2_) resulting in Δ*w*[Ti] is influenced little due to the small content of TiO_2_ (<0.5%) in tundish slag. As a result, a small amount of [Ti] is inevitably oxidized by the stuffing sand along with massive [Al] oxidation. Secondly, [Ti] can be oxidized by oxygen in air and Fe*_x_*O*_y_* in ladle slag and tundish slag during steel transit from the end of RH to the tundish.

**Figure 5:**
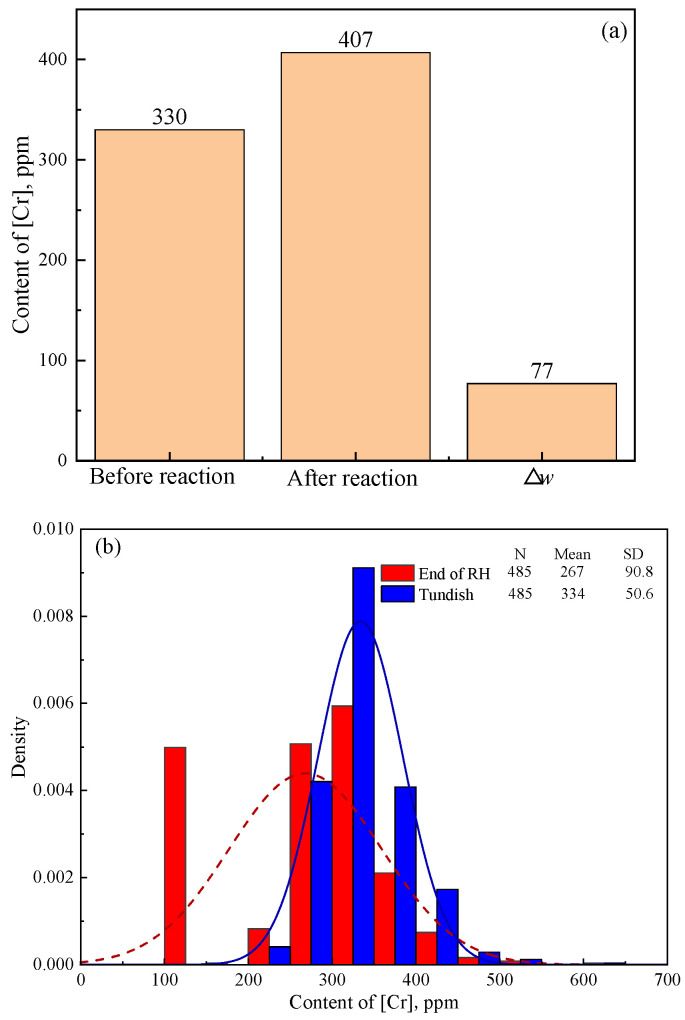
(**a**) Variation in [Cr] content in steel before and after the reaction between stuffing sand and steel based on thermodynamic calculation, and (**b**) variation in the detected [Cr] content from the end of RH to the tundish in industry production.

As the main component of Cr_2_O_3_-based stuffing sand, Cr_2_O_3_ reacts with [Al] as 1/2Cr_2_O_3_ + [Al] → 1/2(Al_2_O_3_) + [Cr], which causes an increase in [Cr] content in IF steel from the end of RH to the tundish. Therefore, the increase in the amount of [Cr] (Δ*w*[Cr]) from the end of RH to the tundish can validate the reoxidation process of IF steel due to the Cr_2_O_3_-based stuffing sand. Figure 5a shows the calculated Δ*w*[Cr] using FactSage 8.0 based on the reaction system in Table 3 and Table 4, and Figure 5b shows the detected [Cr] content of steel samples taken at the end of RH and in the tundish during industry production. The calculated Δ*w*[Cr] in Figure 5a and the average detected Δ*w*[Cr] in practical production in Figure 5b are 77 ppm (407 − 330 = 77) and 67 ppm (334 − 267 = 67), respectively.

The significant increase in the detected [Cr] content from the end of RH to the tundish in Figure 5b validates the reoxidation process of IF steel caused by Cr_2_O_3_-based stuffing sand. The detected Δ*w*[Cr] in Figure 5b is slightly lower than but close to the calculated result in Figure 5a. The detected average Δ*w*[Cr] reaches 87.0% of the calculated one. This indicates that most of the filled stuffing sand reacts with the steel, which results in burning loss of [Al] and [Ti] and thus the pollution of the steel. Meanwhile, the calculated Δ*w*[Al] (92 ppm in Figure 3) is higher than the detected result (62.7 ppm in Figure 2) in industry production. This indicates that the reoxidation of IF steel is primarily caused by the Cr_2_O_3_-based stuffing sand, which is detrimental to the steel cleanliness. The adverse effect of Cr_2_O_3_-based stuffing sand on the steel cleanliness should be even more serious than other factors, like oxygen in air and slag carryover in the last stage of ladle teeming. 

#### 3.1.2. Analysis of Melting Test Results

To further validate the influence of the conventional Cr_2_O_3_-based stuffing sand on the reoxidation of IF steel, a melting test using a vacuum induction furnace was conducted. During the test, 37.2 g of stuffing sand and 117 kg of steel strips were filled in the crucible. The detailed procedure of the melting test has been described in Section 2.2.1. Additionally, the thermodynamic calculation for the melting test was conducted based on Group A of the calculated reaction system in Table 5 and Table 6. The calculation method has been described in Section 2.3.2.

Table 8 shows the detected and the calculated element contents of the original steel strips (before reaction between stuffing sand and steel) and the casting ingots (after the reaction between stuffing sand and steel). The detected and calculated results in Table 8 were obtained based on the melting test and thermodynamic calculation, respectively. Based on the results in Table 8, the burning loss of [Al] and [Ti] (Δ*w*[Al] and Δ*w*[Ti]) and the increase in the amount of [Cr] and [Si] (Δ*w*[Cr] and Δ*w*[Si]) can be determined as follows:

Δ*w*[Al]_Det/Cal_ = [Al]_str_ − [Al]_Det/Cal_ (3)

Δ*w*[Ti]_Det/Cal_ = [Ti]_str_ − [Ti]_Det/Cal_ (4)

Δ*w*[Cr]_Det/Cal_ = [Cr]_Det/Cal_ − [Cr]_str_ (5)

Δ*w*[Si]_Det/Cal_ = [Si]_Det/Cal_ − [Si]_str_ (6)

where Δ*w*[Al]_Det/Cal_ and Δ*w*[Al]_Det/Cal_ represent the detected/calculated burning loss of [Al] and [Ti]; Δ*w*[Cr]_Det/Cal_ and Δ*w*[Si]_Det/Cal_ represent the detected/calculated increase in the amount of [Cr] and [Si]; [Al]_str_, [Ti]_str,_ [Cr]_str,_ and [Si]_str_ represent the element content of the original steel strip; and [Al]_Det/Cal_, [Ti]_Det/Cal,_ [Cr]_Det/Cal,_ and [Si]_Det/Cal_ represent the detected/calculated element content of the casting ingot.

**Figure 6:**
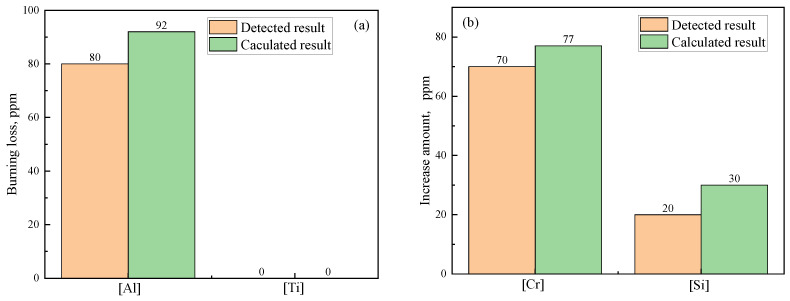
Variation in element content of steel: (**a**) [Al] and [Ti]; (**b**) [Cr] and [Si].

Figure 6 shows the variation in the element content of steel. It can be seen from Figure 6a that obvious burning loss of [Al] occurs due to the Cr_2_O_3_-based stuffing sand and that Δ*w*[Al]_Det_ reaches 80 ppm. However, no burning loss of [Ti] occurs (Δ*w*[Ti]_Mes_ = 0). This is because [Al] can be oxidized by the Cr_2_O_3_-based stuffing sand much more easily than [Ti], as mentioned above in Section 3.1.1. There is a still high content of [Al] (0.024%) in the casting ingot after the reaction. As a result, reoxidation of [Ti] caused by stuffing sand is very difficult. The [Cr] and [Si] content increase with burning loss of [Al] due to Reactions (a) and (b) in Table 7, and Δ*w*[Cr]_Det_ and Δ*w*[Si]_Det_ reach 70 ppm and 20 ppm, respectively, as shown in Figure 6b.

By comparing the calculated burning loss and increase in the amount of [Cr] and [Si] with the corresponding detected results in Figure 6, it can be found that Δ*w*[Al]_Det_, Δ*w*[Si]_Det_, Δ*w*[Cr]_Det_ are 87.0%, 90.9%, 66.7% of the Δ*w*[Al]_Cal_, Δ*w*[Si]_Cal_, Δ*w*[Cr]_Cal_, respectively. Although the detected results are lower than the calculated results, the variation trends of the detected and calculated results are similar. This not only validates the reliability of the calculated tendency but also confirms that Cr_2_O_3_-based stuffing sand is the main factor resulting in burning loss of [Al] and [Ti] during the continuous casting process of IF steel.

### 3.2. Optimization of Stuffing Sand

It is established in the analysis in Section 3.1 that Cr_2_O_3_, SiO_2_, and FeO of the conventional Cr_2_O_3_-based stuffing sand results in the massive reoxidation of steel. In view of this, an attempt was made to develop a new kind of stuffing sand with low oxidability and thus largely decrease the adverse effect of the conventional Cr_2_O_3_-based stuffing sand.

Al_2_O_3_ has stable chemical properties and high temperature resistance. As a result, [Al] and [Ti] in steel is not oxidized by Al_2_O_3_, and the structure of Al_2_O_3_ particles can remain stable when reacting with liquid steel. Considering this, Al_2_O_3_ was adopted to develop a new kind of stuffing sand with low oxidability. Table 2 shows the composition of the potential Al_2_O_3_-based stuffing sand (2#~4#) with low oxidability. PCE tests were conducted to determine the refractoriness of the conventional and the newly developed stuffing sand, and Table 9 shows the results.

As shown in Table 9, the refractoriness of all three Al_2_O_3_-based stuffing sands (2#~4#) is above 1700 °C, which is higher than that of the conventional Cr_2_O_3_-based stuffing sand (1#). This indicates that the refractoriness of all three Al_2_O_3_-based stuffing sands can meet the requirements. SiO_2_ in stuffing sand plays an important role in promoting the stuffing sand to form a sintered layer when reacting with liquid steel. Therefore, a certain amount of SiO_2_ in stuffing sand is necessary. However, as mentioned above, SiO_2_ can cause burning loss of [Al] and [Ti] in steel. For stuffing sands 2#~4#, the SiO_2_ content of 2# is relatively low. Therefore, stuffing sand 2# was chosen as the final scheme.

To validate the effect of the newly developed stuffing sand, a melting test in a vacuum induction furnace was conducted with stuffing sand 2#. The detailed procedure of the melting test has been described in Section 2.2.1. Meanwhile, the thermodynamic calculation for the melting test was conducted based on Group B of the calculated reaction system in Table 5 and Table 6. The detailed calculation method has been described in Section 2.3.2.

Table 10 presents the detected [Al] content of steel based on the melting test and the corresponding calculated results based on the thermodynamic calculation. The detected and calculated burning loss of [Al] are 10 ppm and 14 ppm, respectively, which are significantly lower than the corresponding results for the conventional Cr_2_O_3_-based stuffing sand (Δ*w*[Al]_Det_ = 80 ppm and Δ*w*[Al]_Cal_ = 92 ppm shown in Figure 5). This confirms that the newly developed stuffing sand can largely decrease the adverse effect on the reoxidation of the steel.

**Figure 7:**
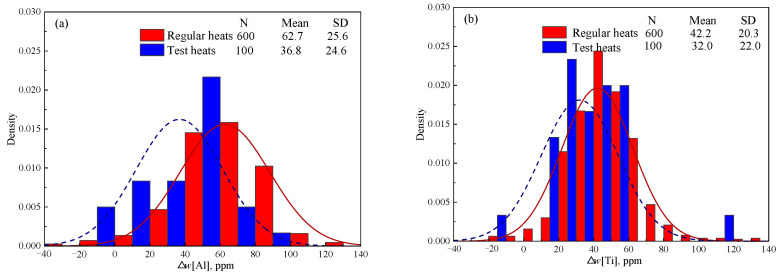
Comparison of (**a**) (Δ*w*[Al]) and (**b**) (Δ*w*[Ti]) between regular and testing heats.

The newly developed Al_2_O_3_-based stuffing sand 2# was subsequently tested in 100 heats of IF steel in the practical industry production, during which the self-opening rate of the ladle was 100%. Burning loss of [Al] and [Ti] for all the testing heats was determined based on the detected [Al] and [Ti] content of the steel samples taken at the end of RH and in the tundish. The detection method has been described in Section 2.2.1. Figure 7 compares Δ*w*[Al] and Δ*w*[Ti] of the testing heats with those of the regular heats using the conventional Cr_2_O_3_-based stuffing sand. Compared with the regular heats, the average Δ*w*[Al] of the testing heats decreases by 41.3% from 62.7 ppm to 36.8 ppm, and the average Δ*w*[Ti] of the testing heats decreases by 24.2% from 42.2 ppm to 32.0 ppm.

**Figure 8:**
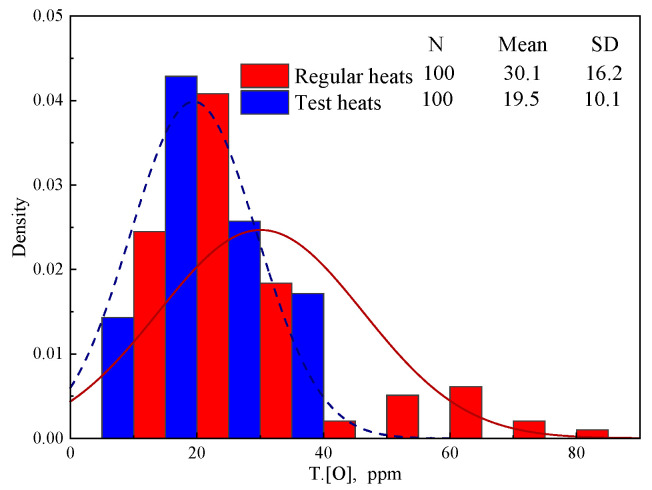
T.[O] of steel samples taken from tundish.

Figure 8 presents the total oxygen content (T.[O]) of the steel samples taken from the tundish. The detection method of T.[O] has been described in Section 2.3.1. Compared with the regular heats, the average T.[O] decreases by 35.2% from 30.1 ppm to 19.5 ppm after the Al_2_O_3_ stuffing sand 2# is applied.

## 4. Conclusions

(1) FeO, Cr_2_O_3_, and SiO_2_ in the conventional Cr_2_O_3_-based stuffing sand can result in the oxidation of [Al] and [Ti] in IF steel, and the oxidability order is FeO > Cr_2_O_3_ > SiO_2_. The [Al] can be oxidized by the stuffing sand more easily than [Ti].
(2) The calculated Δ*w*[Al] can reach 92 ppm for the reaction system of 220 t IF steel and 70 kg Cr_2_O_3_-based stuffing sand when the reaction reaches equilibrium, which is higher than Δ*w*[Al] (62.7 ppm) in the practical production. Cr_2_O_3_-based stuffing sand is one of the main factors resulting in the reoxidation of IF steel.
(3) The measured Δ*w*[Al] (80 ppm) is close to the calculated result (92 ppm) for the melting test in the vacuum induction furnace, and an obvious increase in [Cr] (Δ*w*[Cr] = 70 ppm) from the end of RH to the tundish is found in the industrial production. This further proves that the Cr_2_O_3_-based stuffing sand can result in the reoxidation of IF steel in large scale.
(4) Compared with the conventional Cr_2_O_3_-based stuffing sand, Δ*w*[Al], Δ*w*[Ti], and T.[O] in the tundish are reduced by 41.3%, 24.2%, and 35.2%, respectively, with the newly developed Al_2_O_3_-based stuffing sand applied. This proves that the newly developed Al_2_O_3_-based stuffing sand can largely eliminate the adverse effect of the conventional Cr_2_O_3_-based stuffing sand and further proves that the conventional Cr_2_O_3_-based stuffing sand is one of the main factors resulting in the reoxidation of IF steel.

## Figures and Tables

**Table 1:** Composition of IF steel (M3A35), wt%.

C	Si	Mn	Ti	Al	P	S
0.0030	0.005	0.130	0.060	0.030	0.009	0.006

**Table 2:** Composition of stuffing sand, wt%.

Type	No.	Composition, wt%					
		Cr_2_O_3_	SiO_2_	Al_2_O_3_	FeO	MgO	Residuals
Cr_2_O_3_-based	1#	35.3	20.4	12.2	20.3	8.1	3.7
Al_2_O_3_-based	2#	0.5	8.1	84.7	1.8	1.3	3.6
	3#	0.4	12.3	80.3	1.5	1.2	4.3
	4#	0.6	16.5	76.8	1.6	1.2	3.3

**Table 3:** Composition of IF steel in the calculated reaction system for practical industry production.

Item	Al	Cr	Mn	Si	Ti	Fe	Total
Content, wt%	0.032	0.033	0.130	0.004	0.058	99.7	100
Weight, kg	70.4	72.6	286	8.8	127.6	219,434.6	220,000

**Table 4:** Composition of stuffing sand in the calculated reaction system for practical industry production.

Item	Cr_2_O_3_	SiO_2_	Al_2_O_3_	FeO	MgO	Residuals	Total
Content, wt%	35.3	20.4	12.2	20.3	8.1	3.7	100
Weight, kg	24.71	14.28	8.54	14.21	5.67	2.59	70

**Table 5:** Composition of stuffing sand in the calculated reaction system for melting test.

Group	Item	Cr_2_O_3_	SiO_2_	Al_2_O_3_	FeO	MgO	Residuals	Total
A	Content, wt%	35.3	20.4	12.2	20.3	8.1	3.7	100
	Weight, ×10^−3^ kg	13.13	7.59	4.54	7.55	3.01	1.38	37.2
B	Content, wt%	0.4	12.3	80.3	1.5	1.2	4.3	100
	Weight, ×10^−3^ kg	0.15	4.58	29.87	0.56	0.45	1.60	37.2

**Table 6:** Composition of IF steel in the calculated reaction system for melting test.

Group	Item	Al	Cr	Mn	Si	Ti	Fe	Total
A	Content, wt%	0.032	0.033	0.130	0.004	0.058	99.7	100
	Weight, kg	0.04	0.04	0.15	0.00	0.07	116.65	117
B	Content, wt%	0.032	0.033	0.130	0.004	0.058	99.7	100
	Weight, kg	0.04	0.04	0.15	0.00	0.07	116.65	117

**Table 7:** Gibbs free energy of reaction between stuffing sand and Al and Ti.

Group	No.	Reaction (1550 °C)	ΔG, KJ/mol
A	(a)	1/2Cr_2_O_3_ + Al → 1/2Al_2_O_3_ + Cr	−206.3
	(b)	3/4SiO_2_ + Al → 1/2 Al_2_O_3_ + 3/4Si	−102.6
	(c)	3/2FeO + Al → 1/2 Al_2_O_3_ + 3/2Fe	−316.2
	(d)	3/2MgO + Al → 1/2 Al_2_O_3_ + 3/2Mg	55.1
B	(e)	2/3Cr_2_O_3_ + Ti → TiO_2_ + 4/3Cr	−170.8
	(f)	SiO_2_ + Ti → TiO_2_ + Si	−32.6
	(g)	2/3Al_2_O_3_ + Ti → TiO_2_ + 4/3Al	109.0
	(h)	2FeO + Ti → TiO_2_ + 2Fe	−317.3
	(i)	2MgO + Ti → TiO_2_ + 2Mg	177.8
C	(j)	2Al + Cr_2_O_3_ → Al_2_O_3_ + 2Cr	−412.7
	(k)	1.5Ti + Cr_2_O_3_ → 1.5TiO_2_ + 2Cr	−256.2

**Table 8:** Content of steel, wt%.

Item		Al	Cr	Si	Ti
Steel strip		0.032	0.033	0.004	0.058
Casting ingot	Detected results	0.024	0.040	0.006	0.058
	Calculated results	0.0228	0.0407	0.0070	0.0580

**Table 9:** Refractoriness of different stuffing sands.

Type	No.	Refractoriness, °C
Cr_2_O_3_-based	1#	1610
Al_2_O_3_-based	2#	1740
	3#	1730
	4#	1710

**Table 10:** [Al] content in steel and its burning loss, wt%.

Steel Strip	Casting Ingot		Burning Loss	
	Calculated Value	Detected Value	Calculated Value	Detected Value
0.032	0.0306	0.031	0.0014	0.001

## Data Availability

The original contributions presented in this study are included in the article. Further inquiries can be directed to the corresponding author.
